# PIWIL1 destabilizes microtubule by suppressing phosphorylation at Ser16 and RLIM-mediated degradation of stathmin1

**DOI:** 10.18632/oncotarget.4533

**Published:** 2015-07-15

**Authors:** Chao Li, Xiaoyan Zhou, Jianhui Chen, Yilu Lu, Qianqian Sun, Dachang Tao, Wei Hu, Xulei Zheng, Shasha Bian, Yunqiang Liu, Yongxin Ma

**Affiliations:** ^1^ Department of Medical Genetics, State Key Laboratory of Biotherapy, West China Hospital, Sichuan University, Chengdu, China

**Keywords:** PIWIL1, STMN1, tubulin, proliferation, migration

## Abstract

Human PIWIL1, alias HIWI, is a member of Piwi protein family and expressed in various tumors. However, the underlying mechanism of PIWIL1 in tumorigenesis remains largely unknown. Stathmin1 is a cytosolic phosphoprotein which has a critical role in regulating microtubule dynamics and is overexpressed in many cancers. Here we report that PIWIL1 can directly bind to Stathmin1. Meanwhile, PIWIL1 can up-regulate the expression of Stathmin1 through inhibiting ubiquitin-mediated degradation induced by an E3 ubiquitin ligase RLIM. Furthermore, PIWIL1 can also reduce phosphorylation level of Stathmin1 at Ser-16 through inhibiting the interaction between CaMKII and Stathmin1. Our results showed that PIWIL1 suppresses microtubule polymerization, and promotes cell proliferation and migration via Stathmin1 for the first time. Our study reveals a novel mechanism for PIWIL1 in tumorigenesis.

## INTRODUCTION

Piwi proteins are associated with Piwi-interacting RNAs (piRNAs) [1–3], and involved in stem cell self-renewal, gametogenesis, RNA silencing and transposon control in various organisms [4]. PIWIL1 is a member of the PIWI subfamily defined by the presence of two conserved domains: a PAZ domain in the middle and a PIWI domain in the C-terminal [5]. Recent discoveries have shown that PIWIL1 are involved in cell proliferation and development of various cancers [4]. For example, overexpression of PIWIL1 accelerated growth of human breast and gastric cancer [6, 7], and promoted the migration and invasion of glioma and hepatocellular carcinoma cells [8, 9]. In addition, PIWIL1 was related with poor prognosis of patient in hepatocellular, colorectal, and esophageal squamous *et al* [10–12]. However, the molecular mechanism of PIWIL1 in tumor cells remains to be intangible.

Stathmin1 (STMN1), a cytosolic phosphoprotein, is involved in the regulation of microtubule dynamics [13]. STMN1 has the potential to destabilize microtubule by two identified mechanisms: sequesters α/β-tubulin dimmers, forming a ternary T2S complex; or stimulates microtubule plus end catastrophes [14– 17]. Many studies have confirmed that abnormally high expression of STMN1 is related to human malignancies, and has a positive effect on proliferation, migration and invasion of cancer cells [18–20]. Likewise, high expression level of STMN1 also implied poor prognosis in patient with oral squamous-cell carcinoma and medulloblastoma [21, 22].

Besides, phosphorylation of STMN1 at four serine residues (Ser16, Ser25, Ser38 and Ser63) can impair its microtubule instability activity [23–25]. Subsequent studies demonstrated that PKA phosphorylates Ser16 and Ser63. Moreover, CaMKIV/II (Ca^++^/calmodulin-dependent protein kinase IV/II) in response to Ca^++^ stimulation can affect STMN1 S16 phosphorylation [26, 27]. However, the regulation mechanism of STMN1 phosphorylation is still not well understood.

Here we present that PIWIL1 can bind to STMN1 and form a complex of PIWIL1/STMN1/tubulin. Our current study reveals that PIWIL1 up-regulates STMN1 expression by inhibiting its ubiquitin-mediated degradation dependent on RLIM, and reduces phosphorylation of STMN1 at the Ser16 induced by CaMKII. Thus PIWIL1 inhibits microtubule polymerization and enhances tumor cell proliferation, migration and invasion.

## RESULTS

### PIWIL1 interacts with STMN1

**Figure 1:**
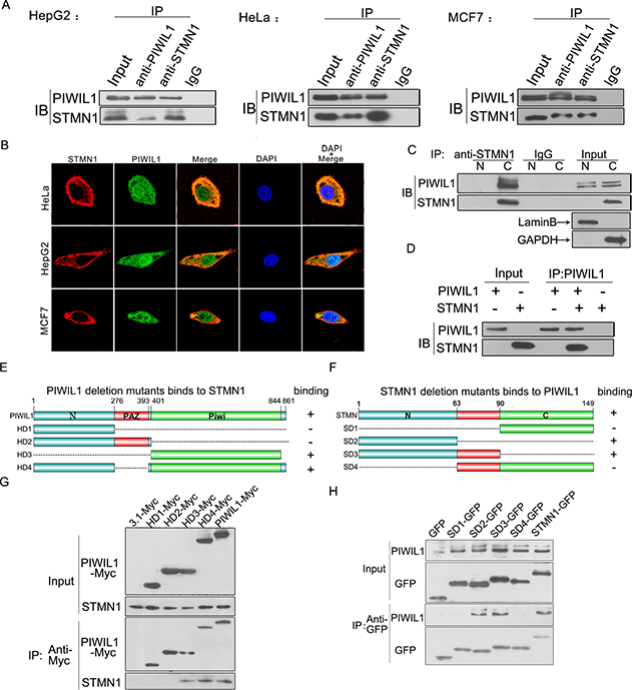
PIWIL1 interacts with Stathmin1 **A.** Endogenous coimmunoprecipitation between PIWIL1 and STMN1 in HeLa, HepG2 and MCF7 cells. **B.** PIWIL1 and STMN1 are colocalized in cytoplasm using immunofluorescence assays. Cells were transfected with HA-tagged STMN1 and Myc-tagged PIWIL1. **C.** Co-localization of PIWIL1 and STMN1 by cytosolic (C)/nuclear (N) fractionation. **D.** TNT^®^Quick Coupled Transcription/Translation System was employed to show that PIWIL1 directly binds to STMN1. **E.** Schematic of PIWIL1 deletion mutants. **F.** Schematic of STMN1 deletion mutants. **G.** Interaction between STMN1 and different MYC-tagged PIWIL1 mutants. **H.** Interaction between PIWIL1 and different GFP-tagged STMN1 mutants.

To better investigate the role which human PIWIL1 protein plays in tumor cells, we performed a bacterial two-hybrid screen using PIWIL1 as the bait and detected STMN1 as a PIWIL1-interacting protein. Coimmunoprecipitation assays were performed and showed that endogenous PIWIL1 and STMN1 can bind with each other in HeLa, HepG2 and MCF7 cell lines (Figure 1A). Immunofluorescence assays showed that endogenous PIWIL1 and STMN1 were mainly overlapped in cytoplasm (Figure 1B). The cytosolic (C)/nuclear (N) fractionation assays further confirmed that the interaction between PIWIL1 and STMN1 mainly occurred in the cytoplasm (Figure 1C). Furthermore, to detect the physical interaction between PIWIL1 and STMN1, TNT Quick Coupled Transcription/Translation Systems *in vitro* were introduced to identify that PIWIL1 directly binds to STMN1 (Figure 1D).

To further investigate the interaction between PIWIL1 and STMN1, we constructed a series of deletion mutants of both proteins to identify the functional domains required for this interaction (Figure 1E and 1F). HeLa cells were transfected with Myc-tagged PIWIL1 mutants. PIWIL1 deletion mutants missing PIWI domain failed to bind to STMN1 (Figure 1G). For better detecting STMN1 mutants, we cloned STMN1 domain mutants into pEGFP-C1 vectors. STMN1 deletion mutants missing N-terminal region failed to bind to PIWIL1 (Figure 1H). Thus, the interaction between these two proteins mainly depends on the PIWIL1 PIWI domain and STMN1 N-terminal region.

### PIWIL1 is involved in up-regulated expression of STMN1 through inhibiting RLIM-mediated degradation

**Figure 2:**
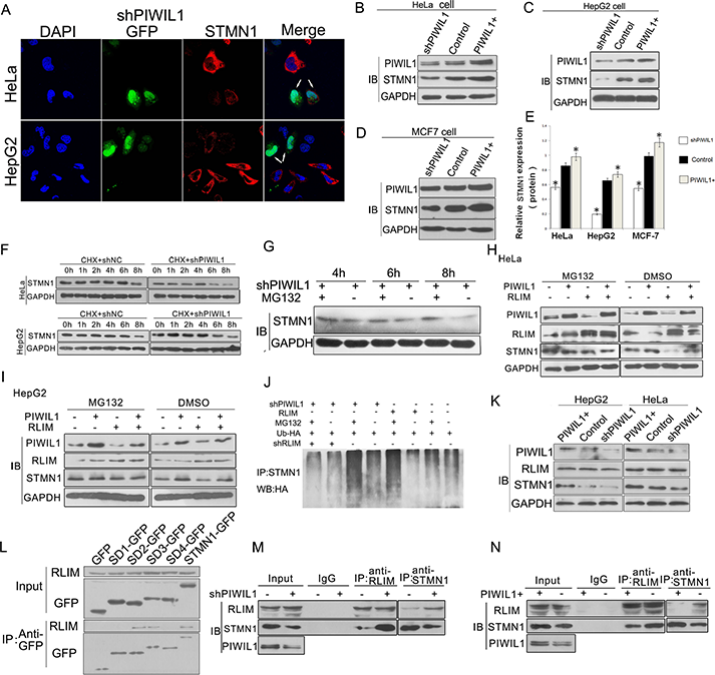
PIWIL1 promotes the expression of STMN1 through blocking RLIM mediated ubiquitin-degradation of STMN1 **A.** Immunofluorescent staining of STMN1 in transfected HeLa and HepG2 cells with STMN1 primary antibodies. The arrows indicate Green fluorescent protein (GFP) expressed from shRNA vectors. **B–E.** PIWIL1 upregulates STMN1 expression at protein level at HeLa, HepG2 and MCF7 cells respectively. These cells were transfected with indicated plasmids and harvested for Western blot analysis. **F.** Stability analysis of STMN1 protein. The HeLa and HepG2 cells transfected with shNC or shPIWIL1 were treated with cycloheximide (CHX) at 50 μM for 0 to 8 h. **G.** MG132 recovered the STMN1 expression reduced by shPIWIL1. **H–I.** PIWIL1 weaken RLIM-mediated-degradation STMN1 at HepG2 and HeLa cells. Cells were transfected with shPIWIL1 alone or in combination with RLIM as shown in the figure, followed by a treatment with MG132 for 6 h. **J.** HeLa cells were transfected with HA-ubiquitin vector, followed with treatment of MG132 for 6 h and precipitated with anti-STMN1 antibodies for Western blot analysis. **K.** Overexpression and knockdown of PIWIL1 lead to no significant change in expression of RLIM in HeLa and HepG2 cells. **L.** Interaction between RLIM and different GFP-tagged STMN1 mutants. **M–N.** PIWIL1 reduces the interaction between RLIM and STMN1. HeLa cells were transiently transfected with PIWIL1 or shPIWIL1 vectors and analyzed by co-immuoprecipitation assays.

Since PIWIL1 interacts with STMN1, it was a matter of interest to check whether PIWIL1 regulate the expression of STMN1. We performed a real-time PCR assay to detect the level of STMN1 mRNA in HeLa and HepG2 cells, and no significant difference was observed after altering PIWIL1 expression (Supplementary Figure S3). Interestingly, PIWIL1 up-regulated STMN1 at protein level (Figure 2A–2E). Treated with cycloheximide (CHX) to inhibit protein synthesis, PIWIL1 knockdown increased the degradation of STMN1 (Figure 2F). Treated with proteasome inhibitor MG132, the downregulation of STMN1 by PIWIL1 knockdown was suppressed, suggesting that PIWIL1 inhibits proteasome degradation of STMN1 (Figure 2G).

Recent study reported that RLIM, an ubiquitin ligase, can influence stability of STMN1 protein and induce its ubiquitin-mediated proteolysis [31]. We assumed that RLIM might participate in the regulation of STMN1 induced by PIWIL1. When PIWIL1 was overexpressed, RLIM can no longer reduce the expression of STMN1 (Figure 2H and 2I). Additionally, ubiquitination assay was performed to show that PIWIL1 knockdown can increase the level of poly-ubiquitination of STMN1. But this phenomenon was eliminated by transfection of RLIM shRNA vector (Figure 2J). We further confirmed that RLIM binds to N-terminal region of STMN1 (Figure 2L). And this interaction between STMN1 and RLIM increased in PIWIL1 knockdowned cells, and decreased in PIWIL1 overexpressed cells without altering the expression of RLIM (Figure 2K, 2M and 2N). These results showed that PIWIL1 can inhibit the ubiquitin-mediated degradation of STMN1 through suppressing the interaction between RLIM and STMN1.

### PIWIL1 inhibits STMN1 phosphorylation at Ser16 induced by CaMKII kinase

**Figure 3:**
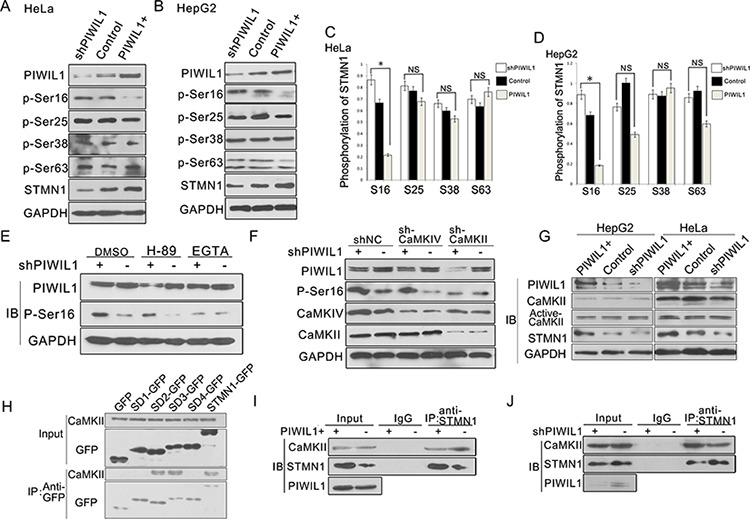
PIWIL1 inhibits STMN1 phosphorylation at Ser16 induced by CaMKII kinase **A–D.** HeLa and HepG2 cells were transfected with shPIWIL1, shNC and PIWIL1 vectors respectively, and analyzed with antibodies against STMN1 phosphorylated at ser16, ser25, ser38 and ser63 respectively. Fold changes were normalized by control to an arbitrary value of one. *, *P* < 0.05. **E.** Hela cells transfected with negative control shRNA or shPIWIL1 were treated with CaMK inhibitor-EGTA (5 mM) or PKA inhibitor-H89 (20 μM) for 2 h. DMSO, dimethylsulfoxide. **F.** shCaMKII blocks PIWIL1 knockdown from upregulating phosphorylation at ser16 of STMN1. HeLa cells transfected with the indicated shRNA were analyzed for protein expression by Western blot. **G.** Overexpression and knockdown of PIWIL1 lead to no significant change in total and active CaMKII in HeLa and HepG2 cells. **H.** Interaction between CaMKII and different GFP-tagged STMN1 mutants. **I–J.** PIWIL1 reduces the interaction between CaMKII and STMN1. HeLa cells were transiently transfected with PIWIL1 or shPIWIL1 vectors and analyzed by immuoprecipitation assays.

The microtubule-destabilizing activity of STMN1 is mainly modulated by phosphorylation at NH2-terminal regulatory domain at ser16, ser25, ser38 and ser63 [32]. Our results showed that PIWIL1 overexpression significantly reduced the level of ser16 phosphorylation in Hela and HepG2 cells (Figure 3A–3D). Previous studies have reported that STMN1 ser16 can be phosphorylated by PKA (cAMP-dependent protein kinase) and CaMKII/IV (calmodulin-dependent protein kinase) [26–28]. To identify the involved kinase, inhibitors of both kinases were introduced. And we found that CaMK kinase inhibitor EGTA, rather than PKA kinase inhibitor H89, eliminated the suppression on Ser16 phosphorylation of STMN1 induced by PIWIL1 (Figure 3E). By using specific shRNAs of CaMKII and CaMKIV, we showed that only CaMKII is involved in Ser16 phosphorylation of STMN1 reduced by PIWIL1 (Figure 3F). Then, the effect of PIWIL1 on total CaMKII and active-CaMKII level was analyzed, but no significant difference was observed (Figure 3G). Co-immunoprecipitation assay further revealed that CaMKII can bind to the N-terminal region of STMN1 (Figure 3H), and this interaction can be suppressed by PIWIL1 (Figure 3I and 3J). These results indicate that PIWIL1 suppresses the phosphorylation at Ser16 of STMN1 through inhibiting the interaction between CaMKII and STMN1.

### PIWIL1 suppresses microtubule polymerization in STMN1-dependent manner

**Figure 4:**
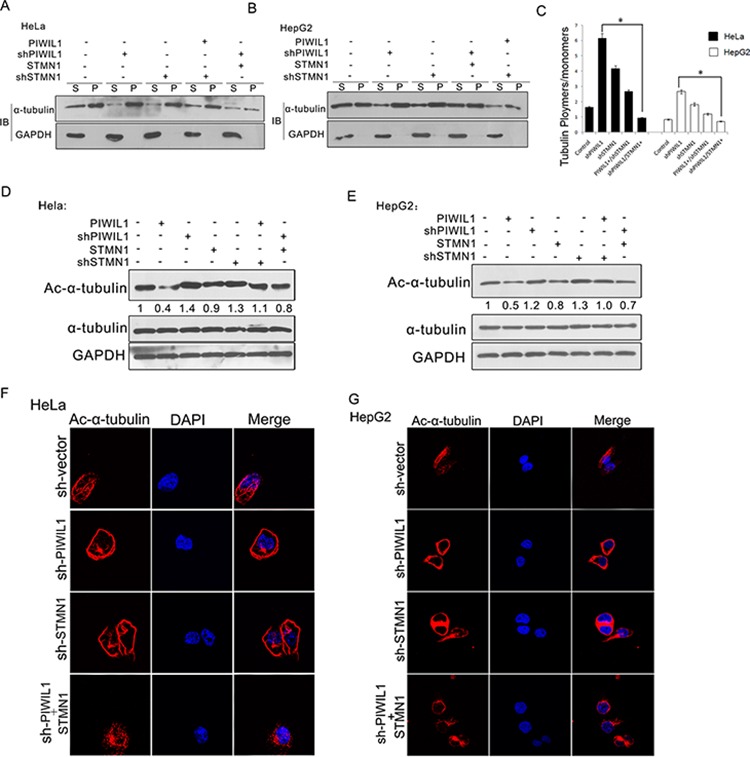
PIWIL1 suppresses microtubule polymerization dependent on STMN1 **A–C.** *In vivo* tubulin polymerization assays in HeLa and HepG2 cells respectively. Supernatant (S) and pellet (P) fractions of cell lysates were analyzed by anti-α-tubulin immunoblotting. **D.** and **E.** HeLa and HepG2 cells transiently transfected with indicated plasmids were analyzed by anti-ac-α-tubulin immunoblotting. **F.** and **G.** shPIWIL1 induces ac-α-tubulin expression in a STMN1-dependent manner using immunofluorescence assays. HeLa (F) and HepG2 (G) cells transfected shPIWIL1 or shSTMN1 vectors and immunofluorescence staining by primary antibodies of anti-ac-α-tubulin.

Previous studies clearly elaborated the microtubule-destabilizing activity of STMN1, so we further performed experiments to examine whether PIWIL1 regulates this process. A microtubule polymerization *in vivo* assay was performed and showed that PIWIL1 overexpression markedly decreased polymerized α-tubulin, and knockdown of PIWIL1 increased polymerized α-tubulin in HeLa and HepG2 cells. However, when STMN1 was overexpressed simultaneously, PIWIL1 knockdown can no longer increase α-tubulin polymerization, and vice versa (Figure 4A–4C). The same changes were observed in the levels of acetylated α-tubulin, which can only be detected in polymerized microtubules (Figure 4D and 4E). Furthermore, laser confocal microscopy (LSCM) was introduced to visually detect the polymerized microtubule using acetylated α-tubulin antibody, showing that knockdown of PIWIL1 enhanced acetylated α-tubulin and polymerized microtubule (Figure 4F and 4G). These results suggested that PIWIL1 suppresses microtubule polymerization in a STMN1-dependent manner.

### PIWIL1 interacts with tubulin to form a PIWIL1-STMN1-tubulin complex

**Figure 5:**
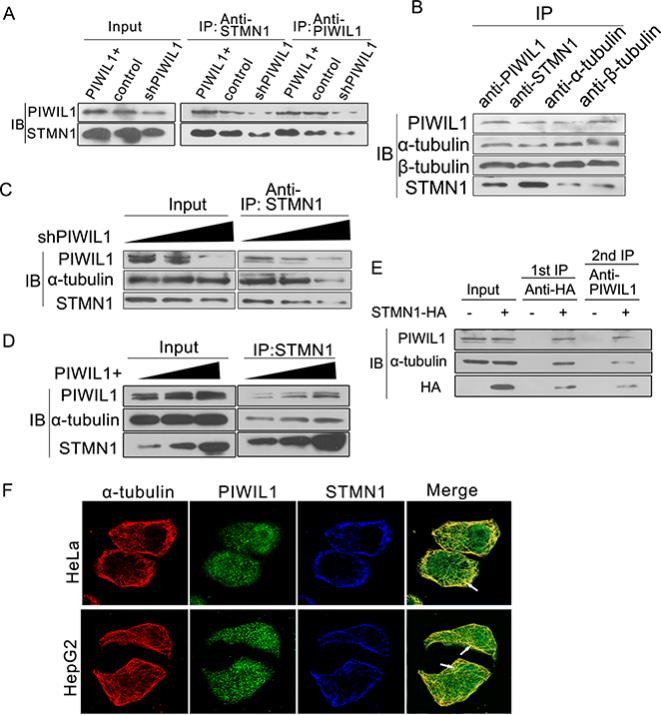
PIWIL1 can enhance formation of PIWIL1- tubulin-STMN1 complex **A.** PIWIL1 increases the interaction between PIWIL1 and STMN1 in HeLa cells. **B.** Co-immunoprecipitation assays showed the interaction of PIWIL1, α-tubulin, β-tubulin and STMN1. **C.** and **D.** PIWIL1 enhances interaction between STMN1 and α-tubulin. HeLa cells were transfected with Myc-PIWIL1 or shPIWIL1 vectors at a concentration gradient (1 μg, 2 μg and 3 μg per well). The lysates were precipitated with anti-STMN1 antibodies by Western blot analysis. **E.** The secondary immunoprecipitation assay with anti-PIWIL1 or IgG. Hela cells were transfected with control vector or HA-STMN1. Cell lysates were immunoprecipitated with anti-HA antibody. HA-STMN1 and the associated proteins were eluted with 4xHA peptide. **F.** Colocalization of α-tubulin, PIWIL1 and STMN1. Cells were transfected with HA-STMN1 vector and harvested for immunofluorsence assay with goat anti-HA (blue), rabbit anti-PIWIL1 (green), and mouse anti-α-tubulin antibodies (red) in HeLa and HepG2 cells respectively. The arrows indicate colocalization of PIWIL1, STMN1 and α-tubulin.

We next showed that the interaction between PIWIL1 and STMN1 increased in PIWIL1 overexpressed HeLa cells (Figure 5A). Endogenous coimmunoprecipitation reveals that PIWIL1 can also bind to α- and β-tubulin (Figure 5B). Furthermore, PIWIL1 can increase the interaction between STMN1 and α-tubulin in Hela cells (Figure 5C and 5D). Taken together, we speculated that PIWIL1, STMN1 and tubulin formed a complex. And this hypothesis was confirmed by a two-step immunoprecipitation assay (Figure 5E). Immunofluorescence assays also showed that PIWIL1, α-tubulin and STMN1 were colocalized mostly in cytoplasm (Figure 5F). Together, these results showed that PIWIL1 binds to tubulin and form a PIWIL1-STMN1-tubulin complex.

### STMN1 mediates PIWIL1-regulated tumor cell proliferation and invasion

**Figure 6:**
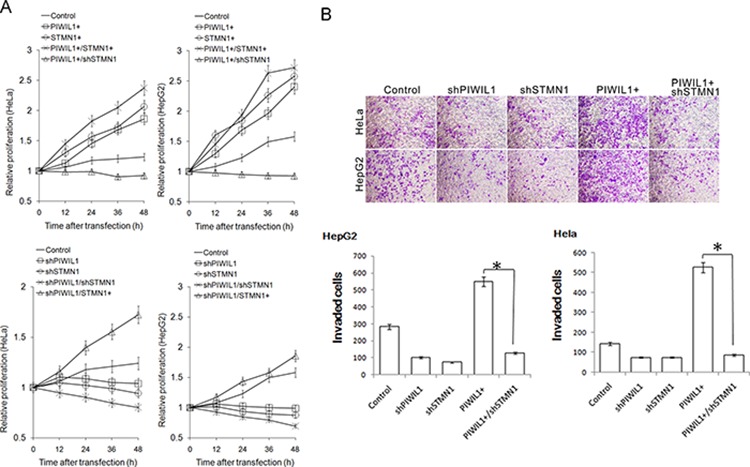
PIWIL1 can enhance cell proliferation and cell invasion via STMN1 **A.** Cell Counting Kit-8 assay indicated that STMN1 knockdown eliminates impact of PIWIL1 on cell proliferation. But STMN1 overexpression recovers impact of PIWIL1 knockdown on cell proliferation. HeLa and HepG2 cells were transfected with indicated vectors. Fold changes were calculated by absorbance and normalized by control to an arbitrary value of one. **B.** PIWIL1 enhances tumor cell invasion through STMN1. HeLa and HepG2 cells were incubated with serum-free medium for 24 h, plated into the top side polycarbonate transwell filter chambers coated with Martigel, and cultured for 12 h. Cells on the upper side of the membrane were then removed and underside cells were fixed, stained with crystal violet.

We surmise whether PIWIL1 promotes tumor cell proliferation and invasion via STMN1. Using cell counting kit-8 assays to observe the cell proliferation in HeLa and HepG2 cells, we found that overexpressed PIWIL1 enhanced cell proliferation in both cell lines, which can be recovered by knockdown of STMN1 and vice versa (Figure 6A). We next detected whether PIWIL1 can regulate tumor cell migration and invasion by performing Transwell assays. Results showed that PIWIL1 overexpression markedly promotes cell migration and invasion, which can be recovered by knockdown of STMN1 and vice versa (Figure 6B and Supplementary Figure S2B). The result that PIWIL1 overexpression promotes the tumor migration was also verified by wound-scratch (healing) assay (Supplementary Figure S2A). These results suggested that PIWIL1 can promote tumor cell migration and invasion through STMN1.

## DISCUSSION

Previous studies have suggested that PIWIL1 plays a significant role in carcinogenesis [10]. However, the mechanism that PIWIL1 plays in carcinogenesis remained unclear yet. To further investigate the signaling pathway through which PIWIL1 regulates in tumor cells, we performed a bacterial two-hybrid assay using PIWIL1 as the bait and identified STMN1 as a target-protein. We further confirmed this interaction using coimmunoprecipitation and immunofluorescence assays (Figure 1). STMN1 can regulate microtubule dynamics through forming a T2S complex with α/β-tubulin, promoting microtubule-depolymerization and preventing α/β-tubulin heterodimers polymerization [33]. As microtubule dynamics is essential for mitosis, cell migration and epithelial mesenchymal transition (EMT) [13], STMN1 plays an important role in tumorigenesis. Previous studies also showed that STMN1 is aberrantly overexpressed in various human tumors [34], and the microtubule destabilizing activity of STMN1 can be regulated in expression and phosphorylation manner [13, 16, 28, 34].

Our research shows that PIWIL1 promotes STMN1 expression at protein level but not mRNA level (Figure 2A–2E and Supplementary Figure S3). Immunopreciptitation assays further demonstrated that PIWIL1 knockdown increased the interaction of STMN1 and RLIM (Figure 2M). We identified that PIWIL1 promotes the expression of STMN1 by attenuating the ubiquitination and degradation of STMN1 induced by RLIM. The microtubule destabilizing activity of STMN1 can be suppressed by kinases such as PKA, MAPK, CaMKII/IV and CDKs through phosphorylation at Ser16, Ser25, Ser38 and Ser63 resides of STMN1 [16, 35]. Our present study uncovers that PIWIL1 suppresses the phosphorylation of STMN1 at Ser16 but not other resides (Figure 3A–3D) through suppressing the interaction between STMN1 and CaMKII (Figure 3I–3J). We further confirmed that PIWIL1 interacts with tubulin and can form a PIWIL1-STMN1-tubulin complex (Figure 5A–5F). Our research shows that PIWIL1 suppresses MT polymerization in STMN1-dependent manner by using microtubule polymerization *in vivo* assay and immunofluorescence analysis (Figure 4A–4G) and further promotes cell proliferation and migration (Figure 6 and Supplementary Figure S2).

**Figure 7:**
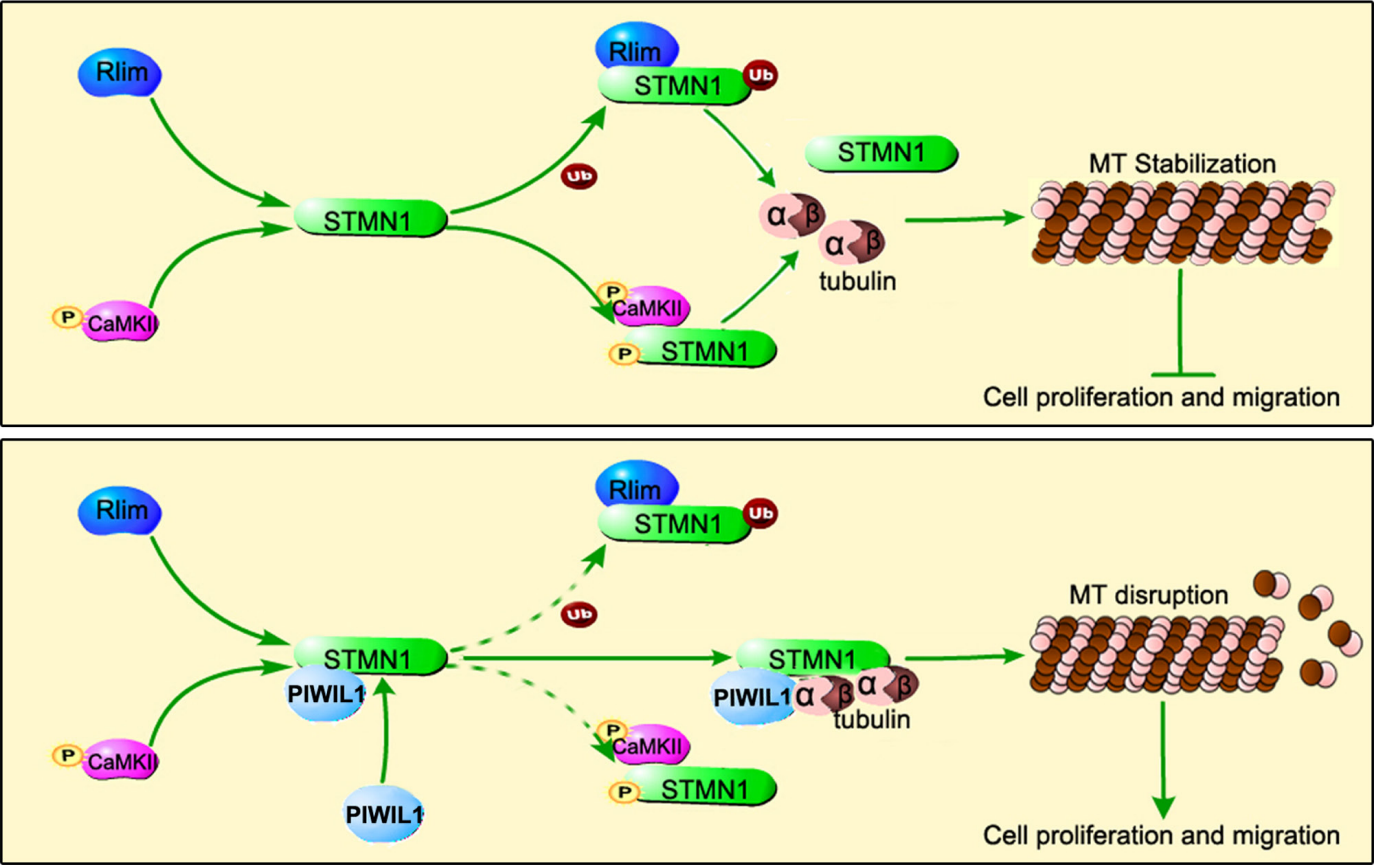
Model of PIWIL1 modulation on microtubule dynamics via STMN1

In summary, our results show that PIWIL1 binds to STMN1 and can form a complex of PIWIL1/STMN1/tubulin, suppressing RLIM mediated ubiquitination and CaMKII mediated phosphorylation at Ser16 of STMN1. The PIWIL1/STMN1/tubulin complex suppresses MT polymerization and thus promotes tumor cell migration and proliferation (Figure 7). Considering that microtubule dynamics contributes to a variety of significant bioprocess, our research provides a new perspective on the role of PIWIL1 protein in cell proliferation and migration, and extends the function of the PIWI protein in tumorigenesis.

## MATERIALS AND METHODS

### Expression vectors, shRNA and antibodies

cDNAs encoding Myc-tagged PIWIL1, HA-tagged STMN1 and HA-tagged RLIM were synthesized and inserted into pcDNA3.1(+) (Invitrogen) vector. Meanwhile, we constructed a series of Myc-tagged PIWIL1 mutants (described in Figure 1E) and EGFP-tagged STMN1 mutants (described in Figure 1F) by segmented-PCR and fusion PCR.

shRNAs for PIWIL1, STMN1, RLIM, CaMKII, CaMKIV and shNC (Negative Control SuperSilevcing shRNA) were synthesized and cloned into pSUPER-pGPH1/GFP/Neo by Shanghai GenePharma Inc. PR China. The sequences of the shRNAs we used were based on references (PIWIL1 shRNA [10], STMN1 shRNA [28], CaMKII shRNA [29], CaMKIV shRNA [30] and RLIM shRNA [31]).

The target sequences of these shRNA were as follows:

PIWIL1 shRNA: 5′-GCCGTTCATACAAGACTA ATT-3′

STMN1 shRNA: 5′-AGGCAATAGAAGAGAAC AA-3′

CaMKII shRNA: 5′-CCACTACCTTATCTTCGAT-3′

CaMKIV shRNA: 5′-GATGGCAACGAGGACAT GA-3′

RLIM shRNA: 5′-GGTCTCAGACACCAAACAA CA-3′

The antibodies used in WB and Co-IP experiments are listed below: Rabbit anti-Ac-α-tubulin, Mouse anti-HA (Cell Signaling Technology); Rabbit anti-PIWIL1, Rabbit anti-STMN1, Rabbit anti-GAPDH (Abcam); Mouse anti-tubulin (Beyotime Biotechnology); Rabbit anti-RLIM, Rabbit anti-GFP, Rabbit anti-CaMKII, Rabbit anti-CaMKIV, Rabbit anti-active CaMKII (Proteintech), Rabbit anti-pS16-STMN, Rabbit anti-Myc, Rabbit anti-HA (Santa Cruz Biotechnology), Rabbit anti-pS25-STMN, Rabbit anti-pS38 (Bioworld Technology), Rabbit anti-pS63-STMN, Mouse anti-Myc (ZSGB-BIO).

### Cell culture and transfection

Human cervical cancer cell line HeLa, hepatocellular carcinoma cell line HepG2 and breast carcinoma cell line MCF7 were maintained in State Key Laboratory of Biotherapy and Cancer Centre of West China Hospital, Chengdu, PR China. Cells were cultured in DMEM with 10% FBS. For western blot analysis, a density of 1–1.5 × 10^5^ cells/ml were seeded 24 h before transfection using jetPRIME™ (Cat. No. 114-15, Polyplus-transfection SA, France) and harvested after 48 h. For RT-PCR analysis, cells were harvested 24 h after transfection. For MG132 treatment, Cells were treated with the proteasome inhibitor MG132 (20 mM) at a final concentration of 10 μM for 6 h before harvesting. For cycloheximide (CHX) treatment, cycloheximide was dissolved and the final concentration was 50 μM.

### Coimmunoprecipitation and western blotting

After cells were transfected with the designated plasmids, cells were harvested into universal protein extraction lysis buffer (Cat. No. PP1801, Bioteke, Beijing, PR China) containing protease inhibitor cocktail (Cat. No. 04693116001, Roche, Basel, Switzerland). Extracted proteins were incubated 1–3 μg target antibody or IgG as negative control overnight, and followed with protein A+G agarose beads (Cat. No. P2012, Beyotime, Jiangsu, PR China) for 2 h. Precipitated proteins were subjected to SDS-PAGE, transferred to PVDF membrane (Cat. No. IPVH00010, Millipore, Massachusetts, USA), and detected with specific appropriate primary antibodies and horseradish peroxidase-conjugated secondary antibodies. Specific proteins were visualized using an enhanced chemiluminescence (ECL) Western blot detection system (Millipore).

### *In vitro* binding assays

For protein interaction analysis, *in vitro* protein binding assays were performed using TNT Quick Coupled *in vitro* transcription/translation system (Cat. No. REFL1171, Promega, Madison, WI, USA) according to the protocol. *In vitro* protein expression assays for PIWIL1 and STMN1 were carried out separately in two reactions using TNT^®^system. 1 μg of each plasmid DNA was diluted in 50 μl and incubated at 30°C for 90 minutes. Followed these reactions, 2 μl of each production was used to detect the protein expression of PIWIL1 and STMN1 by Western blot with specific antibodies. Subsequently, 30 μl of each translated protein was mixed together in 200 μl lysis buffer for CO-IP assays.

### Immunofluorescence staining

Cells cultured in 12-well chamber slides were washed three times with cold 1 × PBS, fixed with 4% paraformaldehyde in PBS for 15 min, permeabilized with 0.5% Triton X-100 in PBS for 10 min, blocked with 1% BSA for 30 min at room temperature, incubated with primary antibodies overnight at 4°C and followed with DyLight 488-labeled, DyLight 594-labeled, or DyLight 350-labeled secondary antibodies for 1 h at room temperature. The cells were mounted with DAPI (Sigma) for nuclear staining and the images were acquired with laser scanning confocal microscope (Olympus).

### *In vivo* tubulin polymerization

The *in vivo* assay of polymerized tubulin was carried out as previous described [28]. Transfected cells were lyzed in microtubule-stabilizing buffer [0.1 M Pipes (pH 6.9), 2 M glycerol, 5 mM MgCl_2_, 2 mM EGTA, and 0.5% Triton X-100] containing 4 μM Taxol. Lysates were centrifugated, and microtubules in the pellet were prepared for Western blot analysis.

### Real-time PCR

Total RNA was prepared using TRIzol (Cat. No. 15596-026, Invitrogen, California, USA) from transfected HeLa and HepG2 cells. Target samples were analyzed by RT-qPCR using KAPA SYBR FAST qPCR Kits (Cat. No. KK4601, KAPABIOSYSTEMS, Boston, USA) in a CFX96 Touch real-time PCR detection system (Bio-Rad).

### Cell migration assays

Transfected HeLa or HepG2 cells were cultured in serum-free medium for 16 h. A linear wounds were created with a pipette tip, then observed after 24 h and 48 h. Images were captured using a fluorescence inverse microscope (Olympus). For cell invasion assays, cell were cultured in serum-free medium for 24 h, plated into the top transwell filter chambers coated with Matrigel (Cat. No. 354230, Corning, New York, USA), and incubated for 12 h at 37°C with 5% CO_2_. Migratory cells were fixed in methanol for 15 min, and stained with crystal violet.

### Statistical analysis

Experiments were repeated three times. Western blot results were analyzed with ImageJ software. Statistical analyses were performed using SPSS version 17.0 (IBM Company, Chicago, IL, USA). Differences between experimental groups were determined using Student’ *t* test. Values of *P* < 0.05 were considered as significant and indicated by asterisks in the figures.

## SUPPLEMENTARY FIGURES


