# Supplementary material for: PIWIL1 destabilizes microtubule by suppressing phosphorylation at Ser16 and RLIM-mediated degradation of stathmin1
Source: Oncotarget. 2015 Jul 15;6(29):27794–804. doi: 10.18632/oncotarget.4533 (PMC4695026; doi:10.18632/oncotarget.4533)
Supplement: Supplementary file 1 [file oncotarget-06-27794-s001.pdf]

SUPPLEMENTARY FIGURES

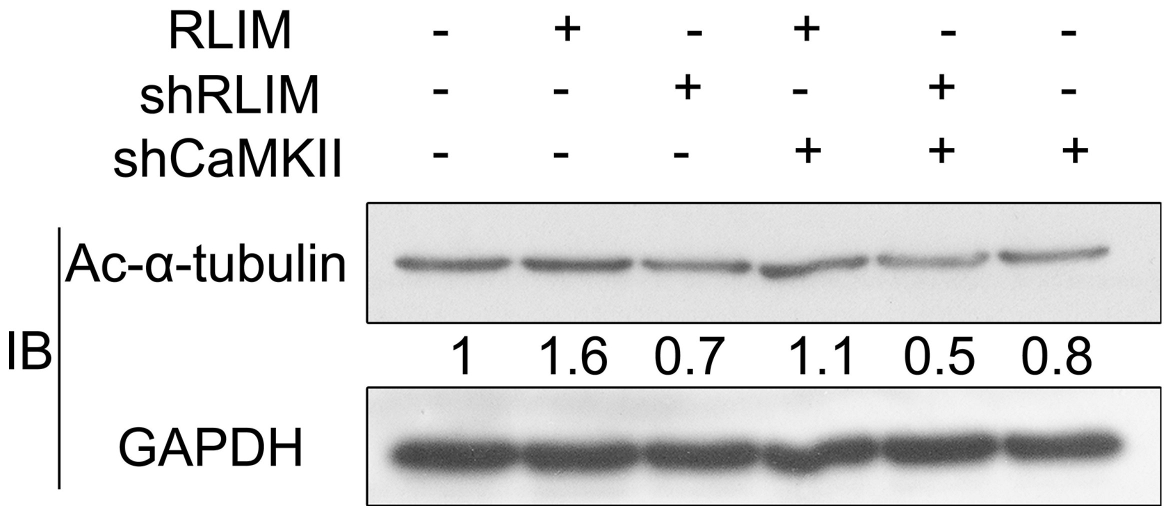

Supplementary Figure S1: RLIM and CaMKII affect microtubule polymerization. HeLa cells transiently transfected with indicated plasmids were analyzed by anti-ac-α-tubulin immunoblotting. Fold changes was normalized by control to arbitrary value of one.

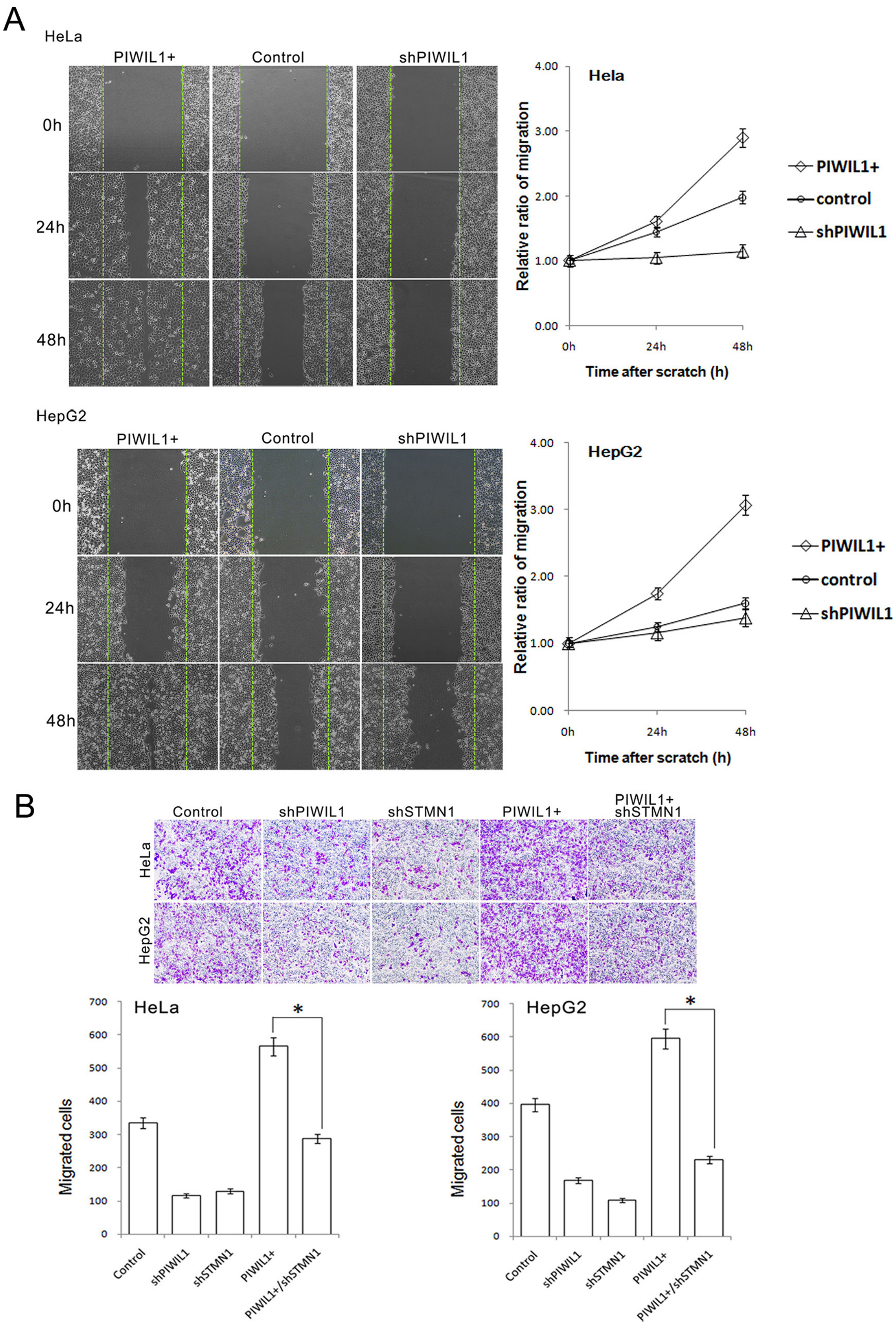

Supplementary Figure S2: PIWIL1 enhances the migration of tumor cells via STMN1. A. PIWIL1 overexpression increases the tumor migration, examined by wound-scratch (healing) assay. The migration of cells was photographed by inverted microscope and the relative migration rate was measurements of the gap size during the culture. **B.** PIWIL1 enhances tumor cell migration via STMN1. Using transwell assay kit with 8 µm pore size polycarbonate inserts, cell numbers were counted in five separate fields by microscopy. The quantified results were presented as the mean ± s.d.

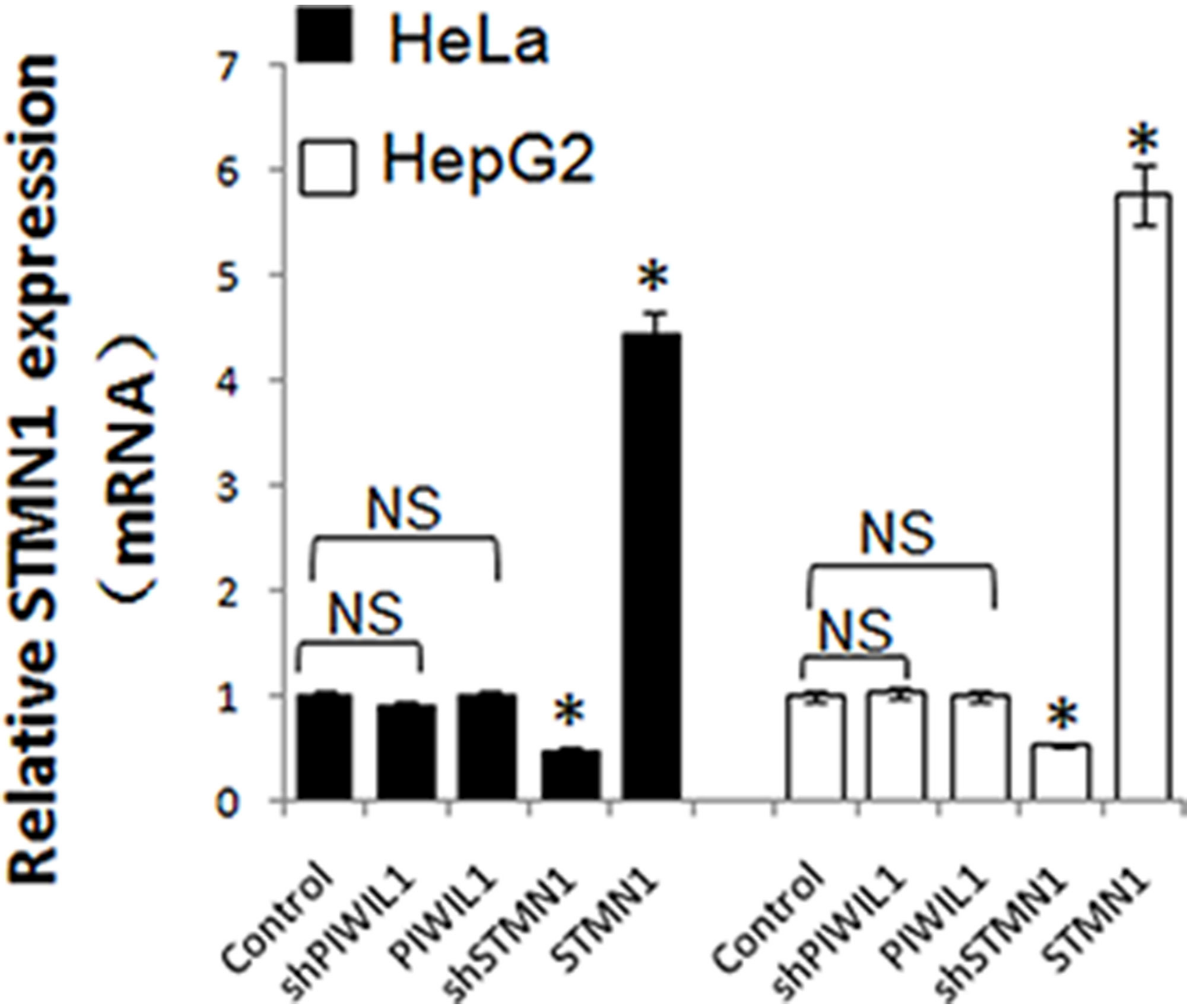

Supplementary Figure S3: PIWIL1 can't change the STMN1 mRNA level. Real-time PCR indicated that the STMN1 mRNA level unchanged after PIWIL1 overexpression or PIWIL1 knockdown. NS, no significant. *, $P < 0.05$.

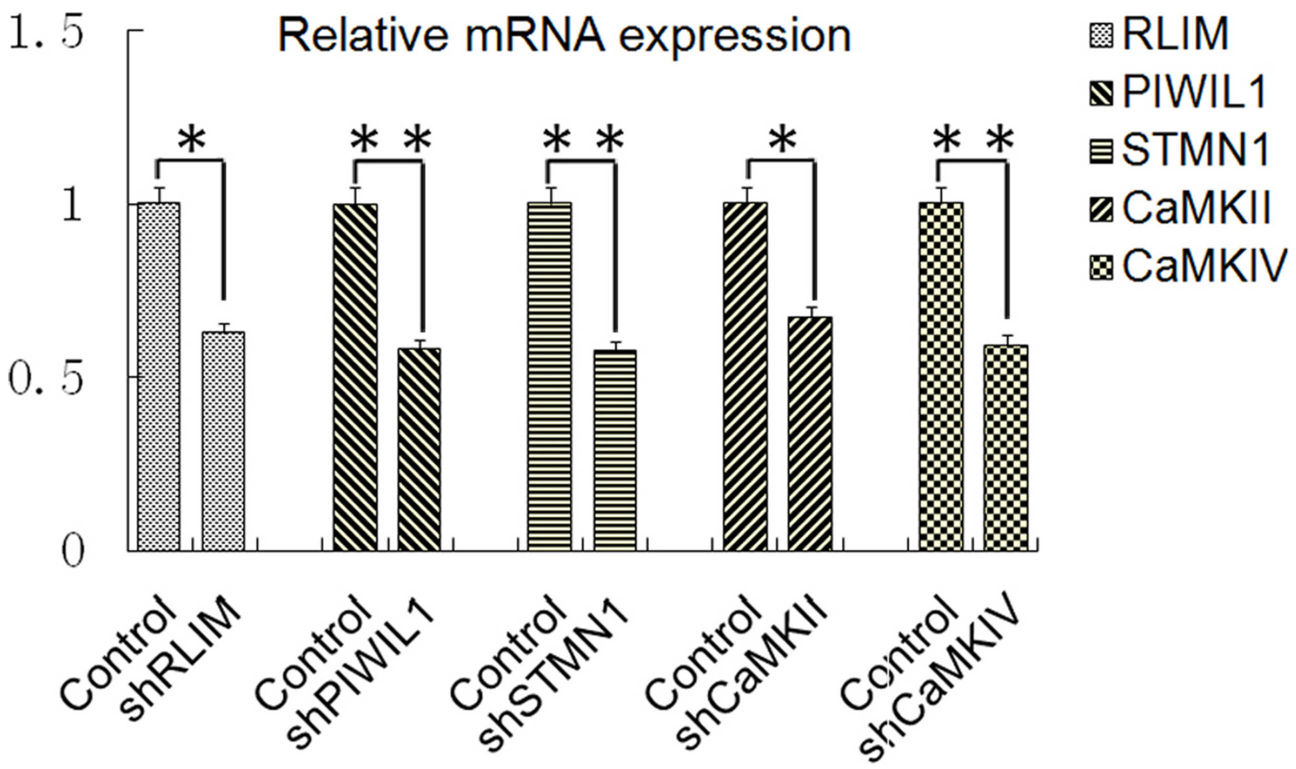

Supplementary Figure S4: The knocking-down efficiency of shRNA. The knocking-down efficiency at mRNA level are verified by Real-time PCR for three repeated experiments. *, $P < 0.05$; **, $P < 0.01$
